# Crazy Farmers

**Published:** 1866-04

**Authors:** 


### CRAZY FARMERS.

Several years ago we made a statement that there were more farmers lunatics than of any other class. Several correspondents of various papers called the truth of the statement in question. We made no reply, as we had the facts in our possession. It is of no use to argue as to the truth of a whole fact; yet many persons are ready at a moment's notice to do this same thing and begin usually to explain away the facts by "supposing a case." We used the fact in order to draw a practical inference of very great value as it regards farmers and farmers' families. The reason given for the fact was that there was too much sameness in a farmer's life; there were too few subjects of thought; the remedy proposed was to increase the intelligence of farmers by directing their attention to the patronage of Agricultural books, magazines, and newspapers; to scientific farming, derided as it is by too many. The time is upon us when only scientific farming will pay; the exhuberant richness of the soil is becoming exhausted in many parts of the country, and in such places a hap-hazard agriculture will not pay in times when labor is doubled and in some cases commands three or four times as much as it used to. See the Journal for January and February of 1863.

The report of the Pennsylvania Hospital for the insane at Philadelphia, made by Dr. Thomas Kirkbride for 1866, as physician-in-chief and superintendent. No similar institution has a more conscientious, faithful and competent head. This last report is of unusual interest and value, on account of its suggestiveness as to the management of such establishments. This report shows that out of four thousand five hundred patients, there were one hundred more farmers than of any other class ; there were more farmers' wives than of any other class, more widows of farmers, than any other class ; and nearly as many farmers' daughters, as of any other class. These things show that, as a general rule, a farmer's life is a hard one, whether for husband, wife or daughter, and we commend the remedy, the effectual remedy proposed in the two numbers of the Journal already referred to, sent post-paid for thirty cents. It is suggestive to note in this connection, that merchants, their wives, daughters and widows are next in number to crowd the insane asylums.

The American Tract Society, 150 Nassau street, New York, offer "Wee Davy," by Norman Macleod, D. D., a beautiful and instructive narrative of Scottish, christian life ; also, "While they are with us," most beautifully suggestive, and which all can read with profit who have any one to love ; the great idea is, treat the living lovingly, and, when they are dead and gone; the reflection will be a balm of sweetest consolation, as long as you live, and growing sweeter to the end. To have a consciousness, abiding alway, that the father, the mother, the child, the wife, now dead, were never neglected, were never treated harshly. Reader, think of it. "How George Neumark sung his hymn." "Individualized Religion," by Dr. Adams, of the Madison Square Church, New-York ; every living christian will be profited and fed by its perusal. "The Power of Truth," by the Rev'd John Gray, a beautiful narrative by a warm-hearted christian man—love divine shines out in every page. "Titles and Attributes of the Holy Spirit," a vest-pocket edition, in Scripture words exclusively ; a precious doctrine, strongly set forth.

A New Sugar.—C. Cory & Sons, of Lima, Indiana, have sent us a good sugar, made from a species of sorghum or sugarcane, from the Sandwich islands ; they can supply the seed or cane. If a barrel of syrup is set aside, more than three-fourths chrystalizes into sugar of itself. These gentlemen have also patented a method of converting the cider or juice of apples into a jam or jelly,which is perfectly delightful ; there is a tartness about it which was a perfect god-send to our suffering soldiers over a year ago, to whom they sent eight thousand pounds. We have some which we have kept for more than a year, without any change ; it is as clear as amber ; a teaspoonful stirred into eight times as much water, dissolves without a particle of sediment and makes a delightful cider. Vacuum pans are not used. The article will soon be largely supplied.

The Richmond, Va., Medical Journal, $5 a year, published monthly, 80 pages 8vo. is edited with great industry, discrimination and ability by Drs. Gaillard and McChesney. Dr. G. has given a most instructive article, with engraving, of Cerebro-Spinal-Meningitis. Dr. Houston contributed an essay on Cholera—for February, which none can read without profit, although in some points there may be a difference of opinion. We heartly commend the Richmond Journal of Medicine to the Profession throughout the country.

VENTILATION. Isaac Pitman of Providence, Rhode Island, has introduced a system of ventilation for *private* dwellings and public buildings, which is well worthy of the attention of builders; he claims for his plan, all the principles in its action, required for a thorough and complete ventilation; producing a state of air indoors, like that without; this certainly has not been accomplished hitherto, and if Mr. Pitman's plan does this, he merits the gratitude of the present and future generations. He aims to give a pure warm air, constantly renewed. Any gentleman who contemplates building or altering a house for his own residence, would do well to communicate with Mr. P.

The Christian Intelligencer of New York says: "Harper's Weekly is the most popular and widely-circulated of American illustrated papers. During the war it manfully and energetically sustained the Government, and still gives it efficient support. Its general reading is good and genial. Terms, $4 a year.

"LAWS OF HEALTH" 427 p. 12mo., by Edward Jarvis, M. D. is just published by A. S. Barnes & Co., 51, 53 & 55 John St., New York, for the use of Schools, Academies and Colleges. The great and sole object of this work is to teach the "laws of Health." It treats of Digestion, Food, Circulation, Nutrition, Respiration, Animal Heat, The Skin, Bones, Muscles, Exercise, Rest, Brain and Nervous System. It is a standard work by an educated man, and written in a style at once clear, practical and instructive to all classes. The publishers have done a public good in issuing this attractive volume, and we hope a demand for it will spring up for copies by the hundreds of thousands, for it well merits it on account of its sterling value and the needs of the times.

